# Supplementary material for: SARS-CoV-2 Vaccine Willingness among Pregnant and Breastfeeding Women during the First Pandemic Wave: A Cross-Sectional Study in Switzerland
Source: Viruses. 2021 Jun 22;13(7):1199. doi: 10.3390/v13071199 (PMC8310322; doi:10.3390/v13071199)
Supplement: Supplementary file 1 [file viruses-13-01199-s001.zip › viruses-1262489-supplementary.pdf]

Supplementary Materials

Table S1. Raw Data used for Figure 2.

		Pregnant Women n=515		Breastfeeding mothers n=1036		Total n=1551	
**Would consider SARS-CoV-2 vaccine**	no at all	123	(23.9)	249	(24.0)	372	(24.0)
	rather no	239	(46.4)	387	(37.4)	626	(40.4)
	rather yes	130	(25.2)	296	(28.6)	426	(27.5)
	yes very	23	(4.5)	104	(10.0)	127	(8.1)
**Influenza vaccine**	No	355/440	(80.7)	642/891	(72.0)	997/1331	(74.9)
	Yes	85/440	(19.3)	249/891	(28.0)	334/1331	(25.1)
**Fear of potential consequences on fetus / infant with influenza**	No	24	(95.3)	897	(86.6)	1388	(89.5)
	Yes	491	(4.7)	139	(13.4)	163	(10.5)
**Usually decline vaccination**	No	418	(81.2)	809	(78.1)	1227	(79.1)
	Yes	97	(18.8)	227	(21.9)	324	(20.9)
**Influenza vaccine is useless**	no at all	489	(95.0)	945	(91.2)	1434	(92.5)
	yes very	26	(5.0)	91	(8.8)	117	(7.5)
**Fear of potential reaction with influenza vaccine**	no at all	515	(100.0)	1534	(99.8)	1549	(99.9)
	yes very	0	(0.0)	2	(0.2)	2	(0.1)
